# Comparative Assessment of Retinal Blood Flow Velocity Changes Following Brimonidine and Brinzolamide Administration Using Retinal Function Imaging

**DOI:** 10.1167/tvst.11.2.1

**Published:** 2022-02-01

**Authors:** Tim J. Enz, Mario Bittner, James R. Tribble, Pete A. Williams, Michael A. Thiel, Martin K. Schmid, Lucas M. Bachmann, Frank Bochmann

**Affiliations:** 1 Department of Ophthalmology, Cantonal Hospital Lucerne, Lucerne, Switzerland; 2 Department of Clinical Neuroscience, Division of Eye and Vision, St. Erik Eye Hospital, Karolinska Institutet, Stockholm, Sweden

**Keywords:** glaucoma, ocular blood flow, retinal blood flow velocity, vasoprotection, neuroprotection, brimonidine, brinzolamide, retinal function imager

## Abstract

**Purpose:**

Impaired ocular blood flow has been associated with the etiopathogenesis of glaucoma. Topical brimonidine lowers intraocular pressure, a major glaucoma risk factor. However, brimonidine's influence on retinal blood flow remains to be fully elucidated. Our aim was to compare the effect of topical brimonidine and brinzolamide administration on retinal blood flow velocity in second and third order vessels in healthy adults using the retinal function imager.

**Methods:**

In 10 healthy probands between 23 and 32 years of age, one eye was randomly selected to receive 2 treatment rounds with 3 single doses of brimonidine 2 mg/mL and brinzolamide 10 mg/mL at 12-hour intervals each. The fellow eyes served as intra-individual controls. Immediately before the first drop and 2 hours after the last drop of each treatment round, all subjects were examined, including Goldmann tonometry, Pascal tonometry, assessment of retinal blood flow velocity using the retinal function imager, as well as blood pressure and pulse measurements.

**Results:**

Intraocular pressure decreased significantly in treated eyes while remaining stable in control eyes, indicating reliable application of brimonidine and brinzolamide drops. In contrast, retinal blood flow velocities did not demonstrate any significant differences between groups after both treatment rounds.

**Conclusions:**

Neither brimonidine nor brinzolamide appear to alter retinal blood flow velocity in a clinically relevant manner. The slight velocity changes detected in our study are likely physiologic fluctuations. Our findings do not support the rationale of a detrimental effect of topical brimonidine on ocular blood flow and hence brimonidine may be further administered for lowering intraocular pressure with the appropriate caution. However, our study is strongly limited by the small sample size and, thus, further research with larger cohorts of healthy volunteers and patients with glaucoma is needed to confirm the results.

**Translational Relevance:**

The study provides information about the effect of the topically administered antiglaucoma medications brimonidine and brinzolamide on the ocular blood flow and its regulation. The findings indicate that beside the lowering of IOP there is no evidence for an additional effect on the development of glaucoma.

## Introduction

Glaucoma is an irreversible neurodegenerative disease characterized by the progressive dysfunction and loss of retinal ganglion cells (RGCs) and their axons, which make up the optic nerve. The disease clinically presents with deterioration of visual sensitivity and progressive visual field deficits. Projected to affect up to 110 million patients worldwide until 2040, glaucoma is the most prevalent irreversible blinding disorder and is a significant health and economic burden.^1^ Age, genetics, and elevated intraocular pressure (IOP) are the major risk factors for glaucoma. Moreover, impaired ocular blood flow has been associated with the etiopathogenesis of the disease.^2^ In order to conserve ocular blood supply and to decelerate the death of RGCs, agents that aim to protect from vascular dysregulation may be of clinical benefit.^3^^–^^7^

Brimonidine is a selective α2-adrenoceptor agonist that lowers IOP by reducing aqueous humor production and by increasing its outflow via the uveoscleral pathway.^8^^,^^9^ The drug is approved for the topical management of primary open-angle glaucoma (POAG) and ocular hypertension (OHT). The mechanism of action is thought to be by activation of the α2-adrenoceptors in the ciliary body, thereby influencing levels of cyclic adenosine monophosphate, which are believed to regulate aqueous humor production.^10^ Furthermore, brimonidine has been suggested to be neuroprotective to RGCs, possibly by modulating neuroinflammatory processes.^11^^,^^12^ Yet, α2-adrenoceptors may also play a role in regulating ocular blood flow (OBF) via constriction of smooth muscle tissue in small blood vessels.^13^^–^^15^ In addition to glaucoma, impaired OBF has been associated with various other ocular diseases, such as age-related macular degeneration and diabetic retinopathy.^16^^–^^20^

Because brimonidine is often administered chronically (years to decades), it is essential to know whether it potentially compromises OBF and consequently contributes to the development or progression of these ocular diseases.^21^ Published evidence is conflicting and although multiple studies using Doppler ultrasonography (DUS), fluorescein angiography (FA), and optical coherence tomography angiography (OCTA) found no evidence for a short-term or long-term effect in healthy volunteers or patients with OHT or glaucoma,^22^^–^^27^ other studies using scanning laser doppler flowmetry (SLDF) or pulsatile OBF measurements as well as animal-based experiments reported signs of possible OBF alterations and retinal vasoconstriction following brimonidine application.^28^^–^^31^ Brimonidine might also influence retinal hemodynamics indirectly by reducing IOP, in addition to any direct effects on the ocular vasculature. In both scenarios, alterations in OBF might be linked to changes in retinal blood flow velocity (RBFV).

To maintain OBF in case of vasoconstriction following brimonidine application, RBFV is expected to increase according to the laws of fluid physics (flow = cross-section area × RBFV). However, RBFV may also increase as a result of decreased vascular resistance following IOP-lowering. To dissect the potential mechanism of brimonidine on OBF and RBFV (stimulation of α2-adrenoceptors or an indirect reduction of IOP), it is necessary to compare the effects of brimonidine to another IOP-lowering medication which does not stimulate α_2_-adrenoceptors. Brinzolamide is a highly specific carbonic anhydrase inhibitor, which lowers IOP by reducing aqueous humor production via decelerating the formation of bicarbonate ions and subsequently reducing sodium and fluid transport in the ciliary bodies.^32^^–^^34^ As such, brinzolamide is not presumed to directly affect retinal vessels. Thus, a comparative analysis of blood flow parameter changes following both brimonidine and brinzolamide application may allow for a conclusion regarding a possible mechanism of action.

Retinal function imaging is a noninvasive diagnostic method that allows for a quantitative mapping of RBFV by direct observation of erythrocyte movement. The retinal function imager (RFI) has proven to be a valuable tool in assessing various retinal conditions and their treatments.^35^^,^^36^ The aim of this study was to explore and compare the effects of topical brimonidine and brinzolamide administration on RBFV in second and third order peri-foveolar vessels in healthy adults using the RFI.

## Methods

This study was conducted in accordance with the Declaration of Helsinki and was approved by the competent ethics committee (Ethikkommission Nordwest - und Zentralschweiz EKNZ, #2015-315). Potential study probands were personally approached and asked to participate. Individuals meeting the inclusion criteria were informed about the study and included when interested. Written informed consent was obtained from all volunteers after explanation of the nature and possible consequences of the study.

As part of this study, IOP was measured by Goldmann applanation tonometry and Pascal tonometry. Within this manuscript, IOP refers to a measurement by Goldmann applanation tonometry unless otherwise stated.

### Inclusion Criteria and Probands’ Characteristics

A total of 10 healthy volunteers over the age of 18 years were enrolled. Probands’ age range was from 23 to 32 years (mean 28.46 ± 2.5 years [± standard deviation]; median 29 years). All study participants were Caucasians. In five individuals, the right eye was selected for treatment (*n* = 5) and in five individuals the left eye was selected for treatment (*n* = 5). All probands were men, as brimonidine is contraindicated in pregnancy and thus female candidates would have had to perform a pregnancy test prior to enrollment. Inclusion criteria comprised: no known hypersensitivity to any of brimonidine and brinzolamide's tartrate's constituents, fully dilating pupils, no signs or history of OHT or other ophthalmic pathology (as assessed upon a clinical examination, including ophthalmoscopy and optical coherence tomography of the macula and the optic disc), no ocular surgery or trauma within ≤3 months, and no severe media opacity or high refractive error (≥+10 dpt or −6 dpt). Furthermore, we did not include individuals with a history of epileptic or any other type of seizure because the RFI uses a stroboscopic flash light, nor probands currently under treatment with monoaminoxidase inhibitors or other antidepressants, in which case brimonidine is contraindicated.

### Clinical Assessment

All data evaluated in this study was collected from four visits per proband on four different evenings (treatment [t]1 to t4) each. Upon the first visit (t1), one eye in each subject was randomly selected to receive 3 single doses of brimonidine 2 mg/mL at 12-hour intervals, whereas the fellow eyes constituted the intraindividual control group. Immediately before the first drop (t1) and 2 hours after the last drop (t2), all subjects were examined, including Goldmann applanation tonometry, Pascal tonometry, and retinal function imaging after pupil dilatation (tropicamide 0.5% drops), as well as arterial blood pressure (BP) and pulse measurement. Mean arterial BP and mean ocular perfusion pressure (OPP; 2/3 of the mean arterial BP minus IOP) were assessed mathematically. The second examination (t2) was conducted 2 hours after the last brimonidine administration as the maximum pharmacological effect is likely to have occurred by this timepoint.

Four to 6 months later (t3), probands were seen again, and the procedure described above was repeated with identical examinations and with treatment of the same eyes, yet with 3 doses of brinzolamide 10 mg/mL drops at 12-hour intervals instead of brimonidine 2 mg/mL. The final examination (t4) was again performed 2 hours after the last brinzolamide administration.

### Retinal Function Imager Measurements

The RFI (Optical Imaging, Rehovot, Israel) has been designed to measure RBFV (mm/s) in the second and third branch retinal vessels, following successful application of the technology for neurovascular imaging of the cerebral cortex.^37^ The operating principle is based on the fact that under green illumination, hemoglobin can be used as a natural, high-contrast chromophore (wavelength 530–590 nm). Thus, images are acquired using a stroboscopic flashlight and a charge-coupled camera. The system is capable of capturing 8 consecutive pictures within no more than 20 ms between the flashes (50–100 Hz), allowing for direct observation of erythrocyte movement. The cross-correlation of moving patterns of erythrocytes and single erythrocytes, respectively, over the eight consecutive pictures renders flow velocity measurement possible. Recording the patient's pulse pattern with a heartbeat synchronization probe attached to a finger or earlobe, flow velocity measurements are standardized for the effect of heart pulsation. With the feasibility of tracking single erythrocytes, even capillaries can be depicted in so called “capillary perfusion maps.” By detecting the direction of blood flow with integrated software, arterioles and venules can be distinguished. Furthermore, by measuring parameters of oxygen utilization and retinal response to photic stimulation, information about oximetry and metabolism can be deducted.^38^^–^^40^ The RFI provides measurements of RBFV for individual vessel segments with a variability of 10.9%.^41^

In our study, RBFV measurements were performed minutes before the first brimonidine (t1) and brinzolamide (t3) application, as well as 2 hours after the last drop applications (t2 and t4). The field of vision was set to 35 degrees. Three series with the best image quality, each with at least four consecutive images, were used for the RBFV analysis. In each retinal hemisphere, 3 arterioles and 3 venules were measured using the automated integrated software, resulting in a total of 12 vessel segment measurements per proband, 6 venules and 6 arterioles. Mean RBFV were calculated based on these 12 measurements.

### Statistical Analysis

Differences in RBFV between interventional and control eyes (t1 to t4) as well as changes between pretreatment (t1 and t3) and post-treatment (t2 and t4) timepoints were defined as primary outcomes. Changes in IOP, mean arterial BP, and heart rate were defined as secondary outcomes. All statistical analysis was performed in R software (R Foundation for Statistical Computing, Vienna, Austria). Descriptive analysis with calculation of mean values and standard deviation was performed. Data were tested for normality with a Shapiro Wilk test. Normally distributed data were compared by a paired Student's *t*-test. Non-normally distributed data were compared by a Wilcoxon signed-rank test. Correlations were assessed by a Pearson's correlation test or Spearman's rank correlation test for normally distributed and non-normally distributed data respectively. Any *P* values < 0.05 was considered statistically significant. For box plots, the center hinge represents the median with upper and lower hinges representing the first and third quartiles; whiskers represent 1.5 times the interquartile range; *P < 0.05, **P < 0.01, ***P < 0.001. Fisher's exact test was performed to compare changes in individual arterial and venous vessel segments before and after treatment with brimonidine and brinzolamide. Frequency distributions based on five equally spaced bins according to the minimum and maximum RBFV values in the total data set (*n* = 60 measurements / cohort / time, minimum = 1.2, maximum = 9.9, bin values: 0 > 2, 2 > 4, 4 > 6, 6 > 8, and 8 > 10) were generated. All vessels (arterioles and venules) before versus after brimonidine (t1 versus t2) and before versus after brinzolamide (t3 versus t4) treatment were tested. There were no significant differences in frequency distributions in any of the interventional groups (see Supplementary Table S1).

## Results

**Figure 1.**
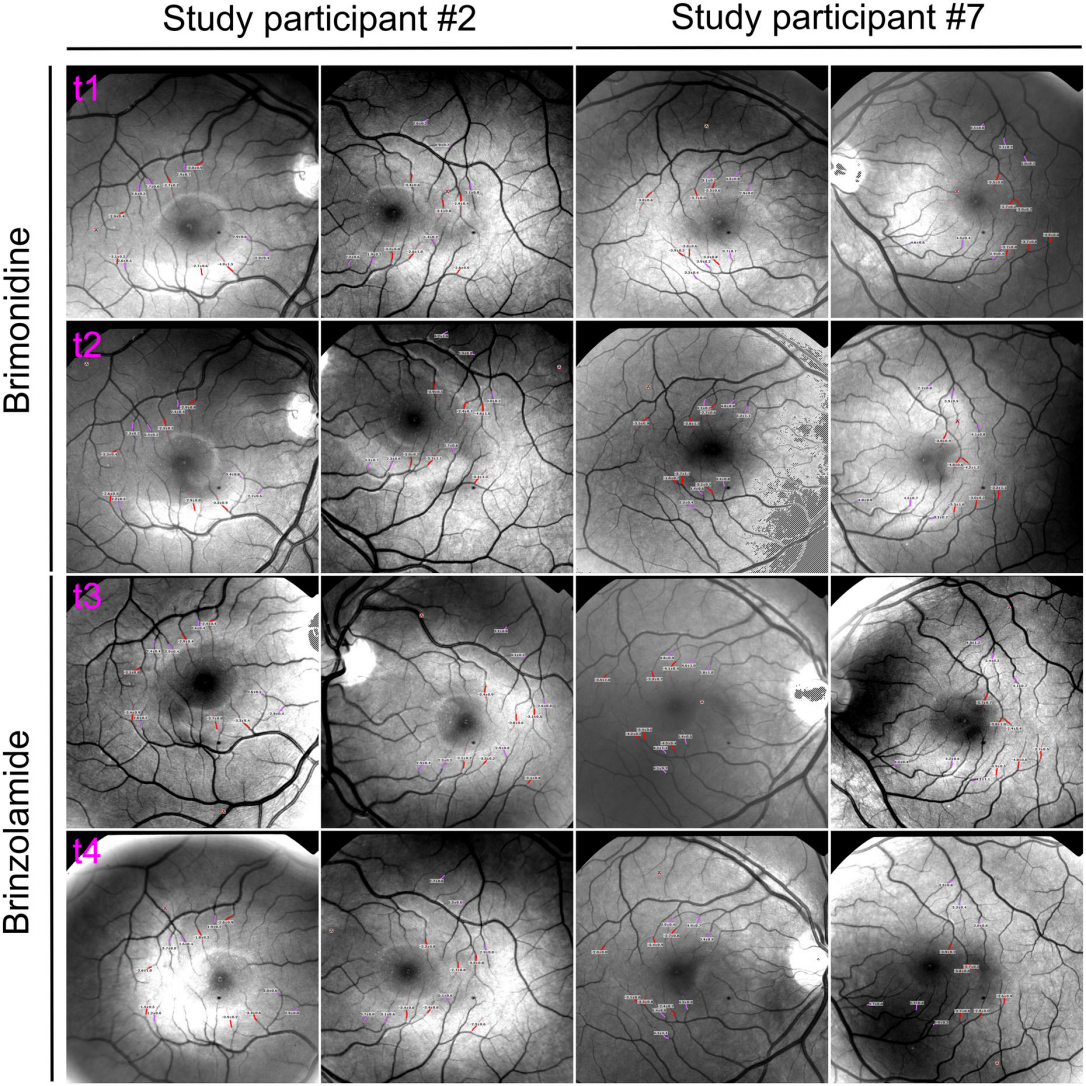
**Exemplary presentation of RFI images with RBFV measurements of two study participants**. *Left columns* (study participant #2): RFI images of study participant #2 before (t1) and after (t2) brimonidine treatment as well as before (t3) and after (t4) brinzolamide treatment, with the left eye being the control eye and the right eye being the interventional eye. *Right columns* (study participant #7): RFI images of study participant #7 before (**t1**) and after (**t2**) brimonidine treatment as well as before (**t3**) and after (**t4**) brinzolamide treatment, with the right eye being the interventional eye and the left eye being the control eye.

Exemplary presentation of RFI images with RBFV measurements of two study participants (#2 and #7) are shown in Figure 1. Additionally, all measured RBFV and corresponding vessel type and order as well as RBFV differences during both treatment rounds (t2 versus t1 and t4 versus t3) of the same two study participants are shown in Table 1.

**Table 1.** Exemplary Presentation of all Measured RBFV and the Corresponding Vessel Type and Order as Well as RBFV Differences During Treatment Rounds (t2 versus t1 and t4 versus t3) of Two Study Participants (#2 and #7)

#2		Control Eye (Left)												Interventional Eye (Right)											
	Vessel #	1	2	3	4	5	6	7	8	9	10	11	12	1	2	3	4	5	6	7	8	9	10	11	12
	Vessel type	a	a	a	a	a	a	v	v	v	v	v	v	a	a	a	A	a	a	v	v	v	v	v	v
	Vessel order	2	3	2	3	2	3	3	3	2	3	3	3	3	3	3	3	3	3	3	3	2	3	2	3
	RBFV t1	3.4	3.1	2.9	3.3	2.6	2.6	2.6	2.9	3.1	2.2	1.9	1.4	2.9	2.7	3.0	3.1	2.7	4.9	2.6	2.7	2.9	2.6	2.9	2.9
	RBFV t2	3.9	2.4	4.6	3.0	3.7	3.4	4.2	3.3	3.0	3.3	2.3	2.7	3.3	2.3	2.9	2.6	2.9	3.5	2.3	3.5	2.4	2.2	3.4	2.7
	Δ RBFV t2-t1	0.5	−0.7	1.7	−0.3	1.1	0.8	1.6	0.4	−0.1	1.1	0.4	1.3	0.4	−0.4	−0.1	−0.5	0.2	−1.4	−0.3	0.8	−0.5	−0.4	0.5	−0.2
	RBFV t3	3.4	3.8	3.1	3.1	3.5	3.1	3.1	3.1	2.6	2.9	2.3	2.9	3.1	2.9	2.4	3.4	3.7	3.3	2.4	2.9	2.4	2.6	2.6	2.9
	RBFV t4	3.3	2.7	3.5	3.4	3.4	2.3	2.7	3.3	2.9	3.7	3.1	3.1	2.6	1.8	2.6	1.5	3.9	3.4	3.7	1.6	1.9	1.2	3.0	2.9
	Δ RBFV t4-t3	−0.1	−1.1	0.4	0.3	−0.1	−0.8	−0.4	0.2	0.3	0.8	0.8	0.2	−0.5	−1.1	0.2	−1.9	0.2	0.1	1.3	−1.3	−0.5	−1.4	0.4	0.0
#7		Control Eye (Right)												Interventional Eye (Left)											
	Vessel #	1	2	3	4	5	6	7	8	9	10	11	12	1	2	3	4	5	6	7	8	9	10	11	12
	Vessel type	a	a	A	a	a	a	v	v	v	v	v	v	a	a	a	a	a	a	v	v	v	v	v	v
	Vessel order	3	3	3	2	3	3	3	2	3	2	3	2	3	2	3	3	2	3	3	3	3	2	3	2
	RBFV t1	3.0	3.7	3.5	3.9	3.8	3.3	3.1	4.3	2.9	3.3	3.9	3.7	3.5	2.7	3.0	3.7	3.7	2.9	3.1	3.1	3.0	4.6	4.5	2.9
	RBFV t2	3.5	3.8	2.7	1.6	3.7	3.1	3.1	4.8	3.3	3.3	3.4	3.4	3.8	4.8	4.2	5.3	3.0	3.3	2.7	3.9	4.1	4.8	4.5	3.1
	Δ RBFV t2-t1	0.5	0.1	−0.8	−2.3	−0.1	−0.2	0.0	0.5	0.4	0.0	−0.5	−0.3	0.3	2.1	1.2	1.6	−0.7	0.4	−0.4	0.8	1.1	0.2	0.0	0.2
	RBFV t3	3.5	3.3	4.1	4.2	3.4	4.5	3.8	4.6	3.8	4.1	4.1	5.4	3.7	3.8	2.4	3.9	4.8	2.7	4.3	3.4	4.1	3.3	4.2	4.1
	RBFV t4	2.0	3.4	2.7	3.1	3.5	2.4	2.9	4.9	3.4	4.1	5.4	4.3	3.9	5.8	2.7	2.7	2.9	2.6	3.5	3.3	2.6	3.7	2.7	3.0
	Δ RBFV t4-t3	−1.5	0.1	−1.4	−1.1	0.1	−2.1	−0.9	0.3	−0.4	0.0	1.3	−1.1	0.2	2.0	0.3	−1.2	−1.9	−0.1	−0.8	−0.1	−1.5	0.4	−1.5	−1.1

a, arteriole; v, venule; RBFV, retinal blood flow velocity in mm/s; Δ RBFV, difference in retinal blood flow velocity in mm/s.

### Brimonidine Treatment Lowers Intraocular Pressure Without Relevantly Altering Retinal Blood Flow Velocity

**Figure 2.**
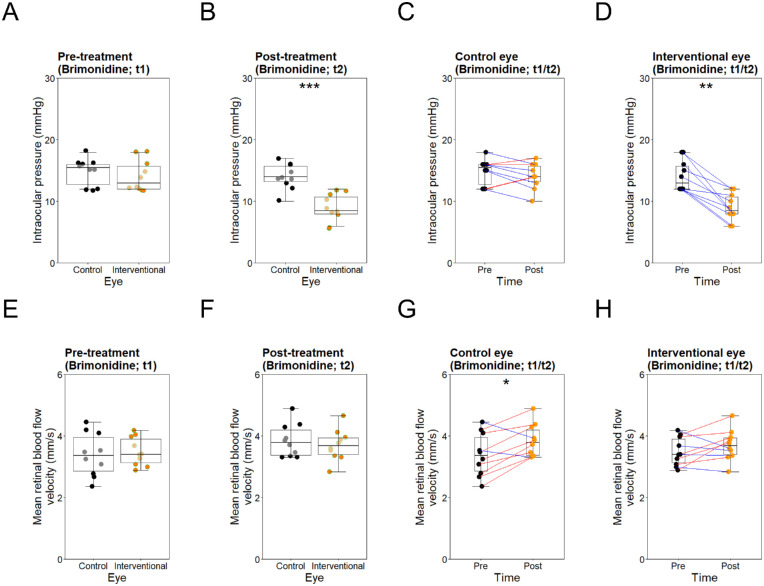
Intraocular pressure and retinal blood flow velocity in the control eyes and the interventional eyes before and after brimonidine treatment. *Top row*: Box and whisker plots showing intraocular pressures (mm Hg) in the control and interventional eyes before (t1) (**A**) and after (t2) (**B**) brimonidine treatment, as well as changes within the control (**C**) and the interventional group (**D**) before (t1) and after (t2) brimonidine treatment. *Red and blue lines* represent increases and decreases, respectively. *P < 0.05, **P < 0.01, ***P < 0.001. *Bottom row*: Box and whisker plots showing overall mean retinal blood flow velocities in the control and interventional eyes before (t1) (**E**) and after (t2) (**F**) brimonidine treatment, as well as changes within the control (**G**) and the interventional group (**H**) before (t1) and after (t2) brimonidine treatment. *Red and blue lines* represent increases and decreases, respectively. *P < 0.05, **P < 0.01, ***P < 0.001. Intraocular pressures and mean retinal blood flow velocities were comparable in both groups before brimonidine treatment. After treatment, intraocular pressures decreased significantly in interventional eyes, whereas remaining stable in control eyes. Blood flow velocities increased slightly in both interventional and control eyes, showing no significant difference between groups.

Before brimonidine treatment (t1), IOP was within normal limits in all eyes (10–21 mm Hg). There was no significant difference comparing control and interventional eyes before treatment for mean IOP (t1; *P* > 0.34), mean ocular pulse amplitude (OPA; *P* = 1), mean OPP (*P* > 0.26), mean arterial RBFV (*P* > 0.54), mean venous RBFV (*P* > 0.66), or mean overall RBFV (*P* > 0.57). Mean arterial BP and mean heart rate were 88.9 (±11.59) mm Hg and 69.4 (±7.14) beats per minute, respectively. Following treatment (t2), mean IOP decreased (−5.1 mm Hg, −36.17%, *P* < 0.05) and mean OPP increased distinctly and significantly (*P* < 0.05) in interventional eyes, whereas remaining stable in control eyes (mean IOP = *P* > 0.24; mean OPP = *P* > 0.06). In both groups, mean overall RBFV increased (interventional eyes: +0.209 mm/s, +5.98%, *P* > 0.18, 95% confidence interval [CI] = −0.123 to –0.543 mm/s; control eyes: +0.461 mm/s, +13.61%, *P* > 0.01, CI 0.106–0.816 mm/s), without demonstrating significant differences between the two groups (*P* > 0.47). The changes in mean arterial RBFV and mean venous RBFV in each group were relatively similar. Mean arterial BP was 95.1 (±5.55) mm Hg and mean heart rate was 63.4 (±8.19) beats per minute. No correlation was found among mean RBFV and mean arterial BP, mean heart rate, mean IOP, or mean OPP post-treatment (all *P* > 0.25). Ophthalmic parameters before and after brimonidine treatment are shown in Table 2 and Figure 2.

**Table 2.** Ophthalmic Parameters in Control and Interventional Eyes Before (t1) and After (t2) Brimonidine Treatment

	Control Eyes	Interventional Eyes	*P* Values Control Versus Interventional Eyes
**Pre-brimonidine (t1)**			
**Intraocular pressure (Goldmann; mm Hg)**			
Mean	14.8	14.1	*P* = 0.3428
Standard deviation	1.99	2.385	
Median	16	13	
Range	12–18	12–18	
**Intraocular pressure (Pascal; mm Hg)**			
Mean	16.72	16.930	*P* = 0.8206
Standard deviation	1.969	1.951	
Median	17	17	
Range	13.8–20.5	14.4–20.1	
**Ocular Pulse Amplitude (Pascal; mm Hg)**			
Mean	2.53	2.53	*P* = 1
Standard deviation	1.019	1.185	
Median	2	2	
Range	1.2–4.4	0.9–5.0	
**Ocular perfusion pressure (mm Hg)**			
Mean	44.444	45.167	*P* = 0.2676
Standard deviation	7.317	7.887	
Median	45	46	
Range	25.333–52.222	25.556–56	
**Overall retinal blood flow velocity (mm/s)**			
Mean	3.388	3.493	*P* = 0.5778
Standard deviation	0.659	0.436	
Med ian	3.358	3.4	
Range	2.367–4.45	2.892–4.183	
**Arterial retinal blood flow velocity (mm/s)**			
Mean	3.680	3.823	*P* = 0.5409
Standard deviation	0.793	0.647	
Median	3.542	3.817	
Range	2.383–5.183	3.017–5.050	
**Venous retinal blood flow velocity (mm/s)**			
Mean	3.097	3.162	*P* = 0.667
Standard deviation	0.581	0.400	
Median	2.925	3.142	
Range	2.350–4.000	2.683–4.000	
**Post-brimonidine (t2)**	(*P* values for intra-group changes between pre and post)	(*P* values for intra-group changes between pre and post)	
**Intraocular pressure (Goldmann; mm Hg)**			
Mean	14.1 (*P* = 0.2449)	9 (*P* = 0.005729)	*P* = 0.0006184
Standard deviation	1.972	2.098	
Median	14	9	
Range	10–17	6–12	
**Intraocular pressure (Pascal; mm Hg)**			
Mean	16.17 (*P* = 0.7507)	12.52 (*P* = 0.0003099)	*P* = 0.01727
Standard deviation	3.792	2.426	
Median	15	12	
Range	9.1–21.9	9.4–17.4	
**Ocular Pulse Amplitude (Pascal; mm Hg)**			
Mean	2.03 (*P* = 0.01767)	1.61 (*P* = 0.008418)	*P* = 0.005121
Standard deviation	0.996	0.902	
Median	2	1	
Range	1–4.4	0.7–3.6	
**Ocular perfusion pressure (Goldmann; mm Hg)**			
Mean	49.3 (*P* = 0.06445)	54.4 (*P* = 0.006485)	*P* = 0.0006184
Standard deviation	4.189	3.724	
Median	49	53	
Range	39–54	48–61.333	
**Overall retinal blood flow velocity (mm/s)**			
Mean	3.849 (*P* = 0.01654)	3.702 (*P* = 0.1879)	*P* = 0.4776
Standard deviation	0.505	0.475	
Median	3.788	3.684	
Range	3.308–4.892	2.833–4.658	
**Arterial retinal blood flow velocity (mm/s)**			
Mean	4.078 (*P* = 0.1248)	3.898 (*P* = 0.7557)	*P* = 0.4767
Standard deviation	0.684	0.672	
Median	4.167	3.983	
Range	3.067–5.150	2.850–4.983	
**Venous retinal blood flow velocity (mm/s)**			
Mean	3.620 (*P* = 0.009298)	3.507 (*P* = 0.05437)	*P* = 1
Standard deviation	0.559	0.548	
Median	3.483	3.492	
Range	3.133–5.050	2.617–4.333	

### Brinzolamide Treatment Lowers Intraocular Pressure Without Relevantly Altering Retinal Blood Flow Velocity

**Figure 3.**
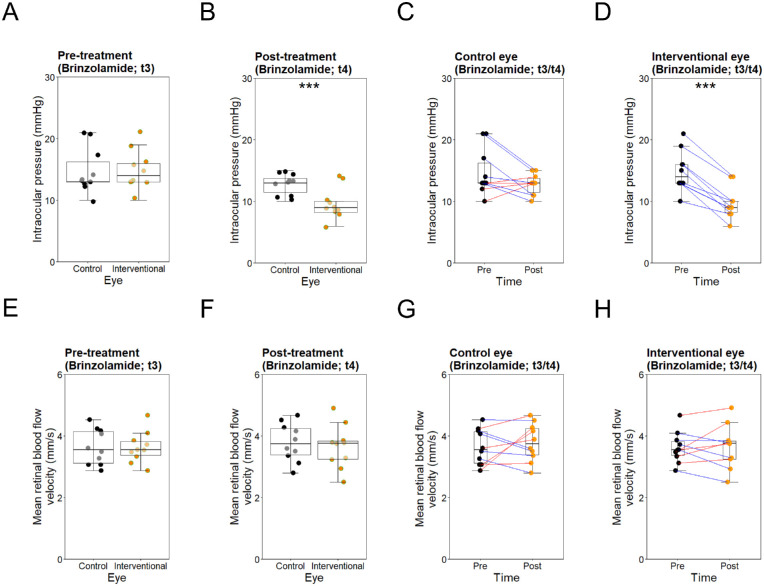
Intraocular pressure and retinal blood flow velocity in the control eyes and the interventional eyes before and after brinzolamide treatment. *Top row*: Box and whisker plots showing intraocular pressures (mm Hg) in the control and interventional eyes before (t3) (**A**) and after (t4) (**B**) brinzolamide treatment, as well as changes within the control group (**C**) and the interventional group (**D**) before (t3) and after (t4) brinzolamide treatment. *Red and blue lines* represent increases and decreases, respectively. *P < 0.05, **P < 0.01, ***P < 0.001. *Bottom row*: Box and whisker plots showing overall mean retinal blood flow velocities in the control and interventional eyes before (t3) (**E**) and after (t4) (**F**) brinzolamide treatment, as well as changes within the control group (**G**) and the interventional group (**H**) before (t3) and after (t4) brinzolamide treatment. *Red and blue lines* represent increases and decreases, respectively. *P < 0.05, **P < 0.01, ***P < 0.001. Intraocular pressures and retinal blood flow velocities were comparable in both groups before brinzolamide treatment. After treatment, intraocular pressures decreased significantly in interventional eyes, while remaining stable in control eyes. Blood flow velocities increased marginally in both interventional and control eyes, showing no significant difference between groups.

Mean IOP was within normal limits before application of brinzolamide (t3), and similar between control and interventional eyes (*P* > 0.78), as well as mean OPA (*P* > 0.10), mean OPP (*P* > 0.69), mean arterial RBFV (*P* > 0.77), mean venous RBFV (*P* > 0.69), and mean overall RBFV (*P* > 0.93). Mean arterial BP and mean heart rate were 93.8 (±3.99) mm Hg and 69 (±8.87) beats per minute, respectively. Following treatment (t4), mean IOP decreased (−5.2 mm Hg, −34.9%, *P* < 0.005) and mean OPP increased (*P* < 0.005) again significantly in interventional eyes, whereas no relevant changes were found in control eyes (*P* > 0.13). Mean overall RBFV increased slightly in both interventional eyes (+0.019 mm/s, +0.52%, *P* > 0.9, CI = −0.323 to –0.361 mm/s) and control eyes (+0.148 mm/s, +4.07%, *P* > 0.5, CI = −0.333 to –0.63 mm/s), showing no significant differences between groups (*P* > 0.19). The changes in mean arterial RBFV and mean venous RBFV in each group were relatively similar. Mean arterial BP was 92.7 (±5.02) mm Hg and mean heart rate was 59.6 (13.71) beats per minute. No correlation was found among mean RBFV and mean arterial BP, mean heart rate, mean IOP, or mean OPP post-treatment (all *P* > 0.25). Ophthalmic parameters before and after brinzolamide treatment are shown in Table 3 and Figure 3.

**Table 3.** Ophthalmic Parameters in Control and Interventional Eyes Before (t3) and After (t4) Brinzolamide Treatment

	Control Eyes	Interventional Eyes	*P* Values Control Versus Interventional Eyes
**Pre-brinzolamide (t3)**			
**Intraocular pressure (Goldmann; mm Hg)**			
Mean	14.7	14.9	*P* = 0.7835
Standard deviation	3.551	3.081	
Median	13	14	
Range	10–21	10–21	
**Intraocular pressure (Pascal; mm Hg)**			
Mean	14.870	15.64	*P* = 0.3647
Standard deviation	2.122	2.36	
Median	15	16	
Range	10.7–18.5	10.5–19.2	
**Ocular Pulse Amplitude (Pascal; mm Hg)**			
Mean	1.9	2.07	*P* = 0.1088
Standard deviation	0.548	0.701	
Median	2	2	
Range	1–2.9	1.2–3.5	
**Ocular perfusion pressure (mm Hg)**			
Mean	47.811	47.611	*P* = 0.6926
Standard deviation	3.61	3.563	
Median	48	49	
Range	40.778–52.667	40.778–52.667	
**Overall retinal blood flow velocity (mm/s)**			
Mean	3.640	3.628	*P* = 0.9392
Standard deviation	0.546	0.480	
Median	3.554	3.550	
Range	2.883–4.525	2.883–4.675	
**Arterial retinal blood flow velocity (mm/s)**			
Mean	3.768	3.818	*P* = 0.7721
Standard deviation	0.533	0.462	
Median	3.708	3.633	
Range	2.867–4.833	3.133–4.650	
**Venous retinal blood flow velocity (mm/s)**			
Mean	3.512	3.437	*P* = 0.6941
Standard deviation	0.709	0.615	
Median	3.283	3.542	
Range	2.650–4.750	2.633–4.700	
**Post-brinzolamide (t4)**	(*P* values for intra-group changes between pre and post)	(*P* values for intra-group changes between pre and post)	
**Intraocular pressure (Goldmann; mm Hg)**			
Mean	12.8 (*P* = 0.1355)	9.7 (*P* = 1.049e-05)	*P* = 5.344e-05
Standard deviation	1.6	2.41	
Median	13	9	
Range	10–15	6–14	
**Intraocular pressure (Pascal; mm Hg)**			
Mean	15.060 (*P* = 0.8275)	13.550 (*P* = 0.0254)	*P* = 0.0254
Standard deviation	2.109	1.766	
Median	16	13	
Range	10.7–17.6	11.3–16.7	
**Ocular Pulse Amplitude (Pascal; mm Hg)**			
Mean	2.03 (*P* = 0.7984)	1.93 (*P* = 0.2359)	*P* = 0.635
Standard deviation	0.718	0.592	
Median	2	2	
Range	1.2–3.9	1.4–3.5	
**Ocular perfusion pressure (mm Hg)**			
Mean	48.867 (*P* = 0.358)	51.967 (*P* = 0.002336)	*P* = 5.344e-05
Standard deviation	3.221	3.347	
Median	48	52	
Range	42.111–54.778	45.111–58.778	
**Overall retinal blood flow velocity (mm/s)**			
Mean	3.788 (*P* = 0.5035)	3.647 (*P* = 0.9017)	*P* = 0.1998
Standard deviation	0.585	0.664	
Median	3.742	3.758	
Range	2.8–4.675	2.508–4.908	
**Arterial retinal blood flow velocity (mm/s)**			
Mean	4.007 (*P* = 0.404)	3.865 (*P* = 0.8308)	*P* = 0.4637
Standard deviation	0.755	0.795	
Median	4.317	3.817	
Range	2.850–4.933	2.633–5.567	
**Venous retinal blood flow velocity (mm/s)**			
Mean	3.570 (*P* = 0.7866)	3.428 (*P* = 0.9621)	*P* = 0.464
Standard deviation	0.674	0.663	
Median	3.550	3.267	
Range	2.367–4.467	2.383–4.583	

## Discussion

### Main Findings

In this exploratory pilot study, we assessed RBFV changes following brimonidine and brinzolamide application. Although both drugs lower IOP, only brimonidine is presumed to have a direct pharmacological vasoconstrictive effect. Following both treatment rounds, mean IOP decreased significantly in interventional eyes while remaining stable in control eyes, indicating reliable application of brimonidine and brinzolamide drops. In contrast, RBFV increased synchronically in both groups following both treatment rounds. No significant differences in mean RBFV were found between control and interventional eyes at any measurement timepoint (t1 to t4). No correlation was found among mean RBFV and mean arterial BP, mean heart rate, mean OPA, or mean OPP post-treatment (t2 and t4).

We hypothesized that RBFV might rise following IOP-lowering either as a result of a direct pharmacological vasoconstrictive effect (only brimonidine) or via reduction of external vascular resistance (brimonidine and brinzolamide). In our cohort, RBFV increased after treatment with both brimonidine and brinzolamide, which may appear suggestive for an effect mediated by reduction of external vascular resistance. However, as RBFV increased synchronically in interventional and control eyes it seems more likely that these changes have occurred independently from the medications’ influence.

After brimonidine treatment, the increases in RBFV were more pronounced in venules compared to arterioles. If there was a direct pharmacological vasoconstrictive effect, it would be more intuitive to expect RBFV to rise in arterioles rather than venules, given the arterioles’ bigger vasoconstrictive potential. On the other hand, evidence suggests that RBFV correlates with vessel diameter.^42^ In case of reduction of external vascular resistance, it could be hypothesized that retinal venules dilate more pronouncedly, whereas arterioles are autoregulated more strongly (unfortunately the RFI only allows for measurement of RBFV without visualization or assessment of vessel size). However, venous RBFV increased more distinctly than arterial RBFV also in the control eyes. Furthermore, after IOP-lowering with brinzolamide, the opposite was the case in both interventional and control eyes. Again, these findings support the conclusion that the measured RBFV changes occurred independently from any medication's influence. In addition, some of the RBFV changes measured during the treatment rounds (t1 versus t2 and t3 versus t4) were similar to those registered between the visits (t1 versus t3). The second treatment round was conducted multiple weeks after the first treatment round and hence a pharmacological effect at the beginning of the second treatment round as a result of the first treatment round can be ruled out. The most likely explanation for the inter-visit (t1 versus t3) variability seems to be physiologic alterations and because this was similar to some of the changes during the treatment rounds it appears conceivable that those changes were physiologic as well.

It is remarkable that the most distinct rise in RBFV was measured in control eyes (after brimonidine treatment). In this group, we also registered a statistically significant decrease in OPA and an increase in OPP which was borderline significant. Published evidence discards a relationship between OPP and OBF. Presumably, stable blood flow does not rebut the possibility of increased flow velocities, yet, in our study, RBFV and OPP did not correlate. Furthermore, following brinzolamide treatment, OPP rose distinctly and significantly whereas RBFV increased only marginally in interventional eyes.

Considering our findings and interpretations, we deem it likely that in this cohort both brimonidine and brinzolamide did not affect OBF in a clinically relevant manner. It is most credible that the changes in RBFV that could be measured after brimonidine and brinzolamide treatment in both control and interventional eyes represent only physiologic fluctuations. The observation that the RBFV remained mostly stable whereas the OPP changed significantly could be explained by the fact that our measurements were obtained from young and healthy individuals. This could be seen as an indicator for an intact vascular autoregulation. Future research could explore patients with glaucoma with clinical signs of vascular dysregulation in which the findings may be different.

### Results in Context of Pre-Existing Literature

Given the different mechanisms of action and the various localizations of the α2-adrenergic receptors, it has been hypothesized that brimonidine possibly affects OBF through a direct pharmacological vasoconstrictive effect and/or indirectly via reducing IOP and, hence, vascular resistance. However, the influence of brimonidine on retinal hemodynamics remains to be fully elucidated, and published evidence is conflicting. Rolle and colleagues documented an increase in OBF after topical brimonidine application assessed by the Heidelberg Retina Flowmeter in patients with glaucoma.^28^ This effect, however, was only observed shortly (2 hours) after the application of brimonidine. After 30 days, the effect was no longer detectable. Vetrugno et al. documented an increase in pulsatile OBF after brimonidine administration in patients with POAG, as assessed by Goldmann applanation tonometry and the Langham system.^29^ Rosa et al. found a heterogeneous vasomotor response mediated by activation of α2-adrenoceptors in porcine retinal vessels after brimonidine application, whereas vasoconstriction increased with higher brimonidine concentration.^30^ However, evidence discarding a detrimental effect of brimonidine on OBF prevails. Lachkar et al. used DUS for examining OBF in a number of different ocular and periocular vessels before and after brimonidine application in patients with OHT and found no significant blood flow velocity changes.^22^ Similarly, Jonescu-Cuypers et al. found no relevant changes in retrobulbar blood flow velocities and arteriovenous passage time following 2 weeks of brimonidine treatment in healthy volunteers, as assessed by DUS and FA.^23^ Assessment of long-term effect of brimonidine on OBF, also using DUS, showed no significant retrobulbar hemodynamic changes when measured after 3 months of brimonidine treatments compared to Latanoprost and Dorzolamide.^24^^,^^27^ In a study by Carlsson et al. using SLDF, no significant difference in OBF and OBF velocity was found between the brimonidine-treated group and the placebo group.^31^ More recent studies evaluated retinal perfusion after brimonidine application in patients with OHT and normal-tension glaucoma by OCTA, and found no evidence for a significant effect.^25^^,^^26^

Our study is the first to assess OBF using the RFI. Demonstrating no relevant changes in OBF parameters, our findings are consistent with other studies based on modern examination techniques and provide further evidence against a detrimental effect of brimonidine on OBF.

### Strengths and Limitations

The strength of our study is its prospective design and the use of retinal function imaging, an innovative and unique diagnostic method enabling direct observation of erythrocyte movement in the retinal vessels. Furthermore, our study offers exclusive insights into the interaction of IOP-lowering agents and ocular blood flow dynamics because we comparatively assessed the effects of two different IOP-lowering agents with distinguished pharmacological features in the same individuals. Previous studies compared blood flow alterations following IOP-lowering treatment only in different individuals. In contrast, it needs to be emphasized that our study is only exploratory and is strongly limited by the small sample size. Based on this pilot study, only restricted inferences can be made regarding the general population. Furthermore, because the investigated subjects were all healthy volunteers, only restricted conclusions can be drawn on patients with pre-existing ocular pathologies, yet setting the stage for investigating patients with compromised retinal vascular dysregulation. In addition, retinal function imaging allows only for indirect evaluation of OBF by measuring RBFV. A change in RBFV does not necessarily signify a clinically relevant alteration in ocular blood supply. It is conceivable that a reduction of vessel diameter is compensated by higher RBFV and thus ocular blood supply remains sufficient. Furthermore, the observation period was short. No statements can be made regarding the long-term course. Given these limitations, further research is needed to confirm our results.

### Implications for Research

The exact interaction between α2-adrenoceptor stimulation and possible subsequent retinal vasoconstriction as well as IOP-lowering, reduction of external vascular resistance, and subsequent vessel dilation remains to be fully elucidated and warrants further investigation. Unfortunately, the RFI only allows for measurement of RBFV and does not provide direct information on vessel size. We are therefore projecting a sequel study using a multimodal retinal imaging approach with which to assess RBFV and vessel size simultaneously in healthy volunteers treated with brimonidine, possibly by combining RFI with OCTA. Future research may be warranted to explore RBFV changes following brimonidine application in patients with glaucoma.

## Conclusion

We demonstrate that neither brimonidine nor brinzolamide alter RBFV in a clinically relevant manner in our cohort of healthy adults. These findings are consistent with prevailing evidence and recent studies based on other modern examination techniques, reporting no relevant changes in OBF parameters after topical brimonidine administration. Our findings do not support the rationale of a detrimental effect of topical brimonidine administration on OBF. Provisionally, topical brimonidine may continue to be clinically used for managing IOP with the appropriate caution. Given the small sample size and other limitations adherent to our study, further research is needed to confirm our conclusions.

## Supplementary Material

Supplement 1
