# Supplementary material for: Comparative Assessment of Retinal Blood Flow Velocity Changes Following Brimonidine and Brinzolamide Administration Using Retinal Function Imaging
Source: Transl Vis Sci Technol. 2022 Feb 1;11(2):1. doi: 10.1167/tvst.11.2.1 (PMC8819484; doi:10.1167/tvst.11.2.1)
Supplement: Supplement 1 [file tvst-11-2-1_s001.docx]

**Supplementary Table S1.** P-values for comparison of RBFV in individual arterial and venous vessel segments before and after brimonidine and brinzolamide treatment (Fisher’s exact test).

| Comparison | Condition | vessel type | p-Value |
| --- | --- | --- | --- |
|  |  |  |  |
| t1 vs t2 (Brimonidine) | Control eyes | Arterioles | 0.2429 |
| t1 vs t2 (Brimonidine) | Control eyes | Venules | 0.0011 |
| t1 vs t2 (Brimonidine) | Interventional eyes | Arterioles | 0.8077 |
| t1 vs t2 (Brimonidine) | Interventional eyes | Venules | 0.4274 |
|  |  |  |  |
| t3 vs t4 (Brinzolamide) | Control eyes | Arterioles | 0.0853 |
| t3 vs t4 (Brinzolamide) | Control eyes | Venules | 0.7604 |
| t3 vs t4 (Brinzolamide) | Interventional eyes | Arterioles | 0.2756 |
| t3 vs t4 (Brinzolamide) | Interventional eyes | Venules | 0.7443 |
